# Labile Carbon Input Mitigates the Negative Legacy Effects of Nitrogen Addition on Arbuscular Mycorrhizal Symbiosis in a Temperate Grassland

**DOI:** 10.3390/plants14030456

**Published:** 2025-02-04

**Authors:** Sitong Liu, Yuxiao Zhang, Xiaoqian Yu, Meng Cui, Liangchao Jiang, Tao Zhang, Yingzhi Gao

**Affiliations:** 1 Institute of Grassland Science, Key Laboratory of Vegetation Ecology of the Ministry of Education, State Environmental Protection Key Laboratory of Wetland Ecology and Vegetation Restoration, Jilin Songnen Grassland Ecosystem National Observation and Research Station, Northeast Normal University, Changchun 130024, China; 2 State Key Laboratory of Vegetation and Environmental Change, Institute of Botany, Chinese Academy of Sciences, Beijing 100093, China; 3 School of Life Sciences, Hebei University, Baoding 071002, China; 4 Key Laboratory of Grassland Resources and Ecology of Western Arid Desert Area of the Ministry of Education, College of Grassland Science, Xinjiang Agricultural University, Urumqi 830052, China

**Keywords:** nitrogen deposition, labile carbon input, arbuscular mycorrhizal fungi, nutrient cycling, plant–microbe interactions, soil management

## Abstract

Nitrogen (N) deposition and carbon (C) addition significantly influence the dynamics of plant–microbe interactions, particularly altering the symbiotic relationship between plants and arbuscular mycorrhizal fungi (AMF). However, the effects and underlying mechanisms of labile C input on the relationship between AMF and various plant species in a nitrogen-enriched environment remain a knowledge gap. A seven-year field experiment was conducted to examine how six levels of N and three levels of labile C addition impact AMF colonization in four key plant species: *Leymus chinensis* (Trin. ex Bunge) Tzvelev, *Stipa baicalensis* Roshev., *Thermopsis lanceolata* R. Br. and *Potentilla bifurca* Linn. Our results showed that N and C additions exert significantly different effects on the relationship between AMF and various plant species. Labile C addition mitigated historical N negative effects, particularly for *S. baicalensis*, enhancing AMF infection and promoting nutrient exchange under high-N and low-C conditions. The relationship between AMF and both *L. chinensis* and *T. lanceolata* changed to weak mutualism under low-N and high-C conditions, with significant decreases in vesicular and arbuscular abundance. Plant root stoichiometry plays a critical role in modulating AMF symbiosis, particularly under high-N and -C conditions, as reflected in the increased AMF infection observed in *T. lanceolata* and *P. bifurca*. Our findings emphasize the species-specific and nutrient-dependent AMF symbiosis, revealing that targeted C input can mitigate the legacy effects of N enrichment. Effective nutrient management is of crucial importance for ecological restoration efforts in temperate grasslands affected by long-term N enrichment.

## 1. Introduction

Global climate change, especially nitrogen (N) enrichment, have significantly impacted ecosystems, particularly grassland ecosystems, where intricate interactions among plants, soil and microbes are essential for maintaining ecological functions [1,2,3]. N deposition has dramatically increased due to human activities such as fossil fuel combustion and fertilizer use [4,5]. Such increases in N inputs can lead to soil acidification, altered microbial communities and disrupted nutrient cycling, ultimately affecting plant health and ecosystem processes [6,7]. This nutrient input has altered plant–soil dynamics, particularly impacting arbuscular mycorrhizal fungi (AMF)’s symbiosis with plants [7,8]. Given the essential role of AMF in nutrient acquisition and soil structure, understanding how N deposition affects AMF–plant interactions is critical for predicting ecosystem responses to environmental changes.

Grasslands represent one of the largest terrestrial biomes and are characterized by complex and diverse interactions between plant roots and soil microbial communities [9]. Among these interactions, AMF play a key role in nutrient uptake, particularly phosphorus (P) and N [2,10,11]. This mutualistic relationship allows AMF to enhance plant growth and survival in nutrient-poor soils by improving nutrient uptake, while plants provide carbon (C) to the fungi [12,13,14]. AMF also contribute to regulating plant community dynamics, nutrient cycling and maintaining ecosystem stability [8,15,16]. However, globally increasing N deposition has altered these interactions by changing soil nutrient availability [6,7,17,18,19]. Elevated N levels often reduce plant reliance on AMF, weaken the mutualism and disrupt nutrient distribution [20,21,22]. These changes can favor competitive grasses over other species, reduce plant diversity and shift species composition [23,24,25]. Additionally, traits such as plant height can influence AMF responses to N enrichment [26]. These shifts suggest that N deposition may have long-term consequences for AMF’s ecological roles and grassland ecosystem stability [16,27,28,29].

In addition to altering plant–AMF interactions, N deposition also affects plant nutrient reserves and root stoichiometric ratios (C/P, N/P and C/N), which are critical for understanding how plants allocate resources under varying nutrient availability [30,31]. Changes in these ratios can influence C allocation to AMF, modifying the balance of the AMF–plant relationship [32]. For instance, elevated soil N can induce P limitation, leading to shifts in root stoichiometry that affect AMF colonization and function [33]. Such stoichiometric imbalances can have cascading effects on the plant-microbe relationship, leading to changes in AMF community composition, colonization levels and functional efficacy. The nutrient status within plant tissues directly affects the AMF–plant mutualism, influencing whether the relationship remains beneficial or shifts toward parasitism, especially under conditions of nutrient imbalance [2,34].

Long-term N deposition leads to the accumulation of nitrogen in both soils and plant communities, with lasting effects on soil quality and ecosystem stability, even after N inputs are ceased [35,36]. To mitigate the legacy effects of nitrogen enrichment, the addition of C has been proposed as a strategy to alleviate soil nutrient imbalances. The addition of organic C sources stimulates the activity of heterotrophic microbes, which can reduce the availability of inorganic nitrogen [37,38]. The effects of C addition on plant communities are likely mediated through the reduction in nitrogen availability. Since AMF cannot directly absorb organic carbon but rely on their host plants for carbon [17], adding C to the soil to reduce nutrient levels can be used to study the dynamics of AM symbiosis under conditions of lowered nitrogen availability. A moderate reduction in nutrient efficiency may promote AMF colonization, as plants increase their investment in mycorrhizal associations to compensate for reduced nutrient uptake [34]. However, substantial nutrient depletion may severely limit plant growth and their capacity to support AMF, resulting in decreased fungal colonization [14]. Research has shown that, after C addition, AMF colonization consistently decreases, and plant size has a limited impact on AMF colonization, with lower colonization levels being a direct consequence of reduced nutrient availability [39]. Furthermore, exogenous C addition can enhance the conversion of inorganic phosphorus to microbial biomass phosphorus (MBP), improving phosphorus availability [40]. An increase in soil phosphorus availability may reduce plant dependence on AMF, potentially leading to a decrease in AMF colonization. In contrast, long-term moderate C inputs have been shown to support AMF communities in soils, enhancing both fungal diversity and vitality [41]. The presence of exogenous carbon sources such as sucrose has also been found to significantly increase AMF colonization in plant roots [42]. It is important to note that different plant species exhibit varying degrees of dependence on mycorrhizal fungi. Changes in C availability can lead to species-specific differences in AMF colonization, with grasses generally showing lower colonization compared to legumes and forbs [39]. Therefore, the changes in AMF colonization are likely not only a direct result of reduced nitrogen availability but also due to shifts in plant–AMF interactions.

While C addition affects the availability of inorganic nitrogen and phosphorus, which in turn influences AMF colonization and activity, the underlying mechanisms—especially in nitrogen-rich environments—remain insufficiently explored. It is currently unclear how C addition affects plant–AMF symbioses across species with different nutrient strategies and growth forms. This knowledge gap emphasizes the need for further research into how exogenous carbon affects AMF mechanisms under varying nitrogen conditions. This study aims to investigate the effects of terminating long-term N enrichment and subsequent labile C addition on AMF symbiosis across four dominant plant species in a temperate grassland ecosystem. We hypothesize the following: (1) Under low-N and high-C conditions, plant species with different nutrient strategies (e.g., grasses vs. forbs) will show distinct responses in their interactions with AMF, with grasses potentially reducing their reliance on AMF, while forbs may maintain stable or enhanced mutualistic relationships; (2) high N and C inputs will lead to increased AMF colonization in *T. lanceolata* R. Br. and *P. bifurca* Linn., with plant root stoichiometry playing a key role in supporting mutualistic relationships. Our study tests these hypotheses across plant species with varying functional traits and nutrient use strategies to provide a comprehensive understanding of how nutrient additions influence AMF–plant interactions. This research offers valuable insights into nutrient cycling, ecosystem restoration and the management of grassland ecosystems in the face of changing environmental conditions.

## 2. Results

### 2.1. Response of AMF Infection Metrics to N Enrichment and C Addition

The impact of historical N enrichment and subsequent labile C addition on AMF infection metrics, including colonization rate (Figure S2), infection density, vesicular abundance, hyphal abundance and arbuscular abundance, were examined across different plant species. A three-way ANOVA revealed significant main and interactive effects of N deposition, C addition and plant species on these AMF infection metrics. Notably, while most AMF infection metrics showed significant responses, AMF infection density was not significantly affected by C addition alone (Table 1).

**Figure 1:**
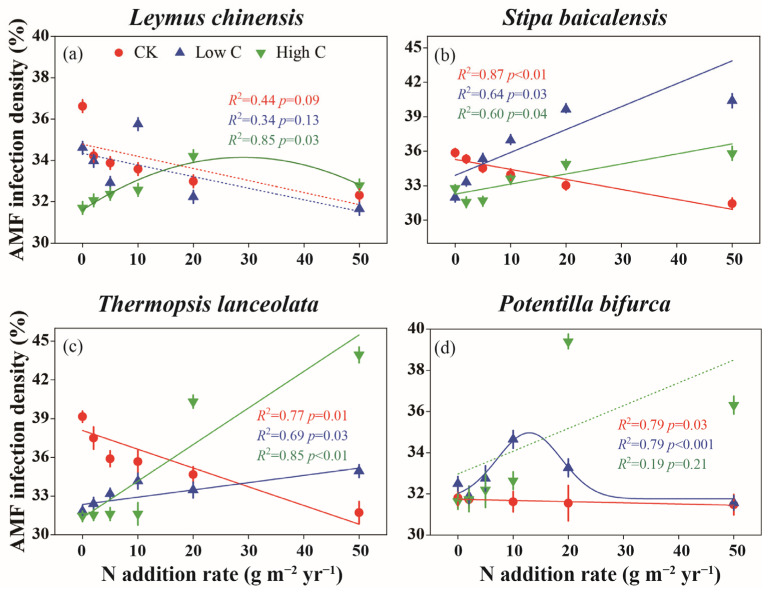
Effects of historical N enrichment and subsequent labile C addition on arbuscular mycorrhizal fungi (AMF) infection density of plant roots across different plant species. (**a**) *L. chinensis* (Trin. ex Bunge) Tzvelev (perennial rhizomatous grass); (**b**) *S. baicalensis* Roshev. (perennial bunchgrass); (**c**) *T. lanceolata* R. Br. (legume); (**d**) *P. bifurca* Linn. (forb). CK represents control treatment with only water added, while Low C and High C correspond to C addition levels of 200 and 2000 g C m^−2^ yr^−1^, respectively. Error bars represent ±SE (n = 8). Data were fitted using linear, polynomial or nonlinear curve fitting methods, selecting the most appropriate curve type for each C level.

In the control treatment (CK), a general decline in AMF infection density was observed as N levels increased across all plant species (Figure 1). For *L. chinensis* (Trin. ex Bunge) Tzvelev, a quadratic relationship emerged between AMF infection density and N addition under high-C condition, with infection density initially increasing at low N levels and then declining as N levels continued to rise (R^2^ = 0.85, *p* = 0.03) (Figure 1a). Low C and high C addition mitigated the negative effects of N on AMF infection density in *S. baicalensis* Roshev. and *T. lanceolata* R. Br., reversing the downward trend typically seen with N enrichment (Figure 1b,c). In *P. bifurca* Linn., low C addition significantly increased AMF infection density at N10 (R^2^ = 0.79, *p* < 0.001), whereas high C addition did not produce a clear linear trend (R^2^ = 0.19, *p* = 0.21) (Figure 1d).

**Figure 2:**
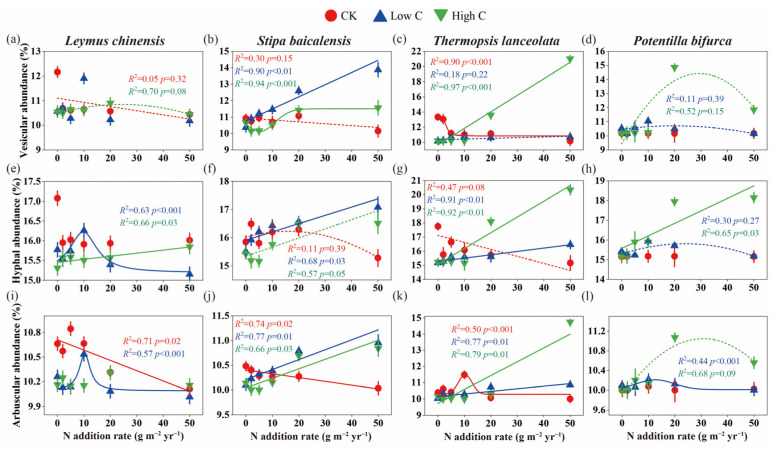
Effects of historical N enrichment and subsequent labile C addition on arbuscular mycorrhizal fungi (AMF) structural abundance in different plant species. (**a**–**d**) Vesicular abundance; (**e**–**h**) Hyphal abundance; (**i**–**l**) Arbuscular abundance. CK denotes control plots with only water added, while Low C and High C correspond to C addition levels of 200 and 2000 g C m^−2^ yr^−1^, respectively. Error bars represent ±SE (n = 8). Data were fitted using linear, polynomial or nonlinear curve fitting methods, with the most suitable curve type chosen for each C level.

Vesicular, hyphal and arbuscular abundance exhibited species-specific responses to N and C treatments (Figure 2). *L. chinensis* showed a significant increase in vesicular abundance at the N10 level with low C addition, while under high-C conditions, vesicular abundance displayed a non-significant quadratic response with increasing N (Figure 2a). In contrast, *S. baicalensis* experienced significant increases in vesicular, hyphal and arbuscular abundance at higher N levels under both low- and high-C conditions (Figure 2b,f,j). For *T. lanceolata* and *P. bifurca*, high-C treatment significantly enhanced vesicular, hyphal and arbuscular abundance at higher N levels (Figure 2c,d,g,h,k,l).

### 2.2. Effects of N and C Additions on Soil Hyphal Length Density

Two-way ANOVA results showed significant effects (*p* < 0.05) of both C and N addition alone and their interactions on the surface soil hyphal length density (HLD) of arbuscular mycorrhizal fungi (Table 2).

**Figure 3:**
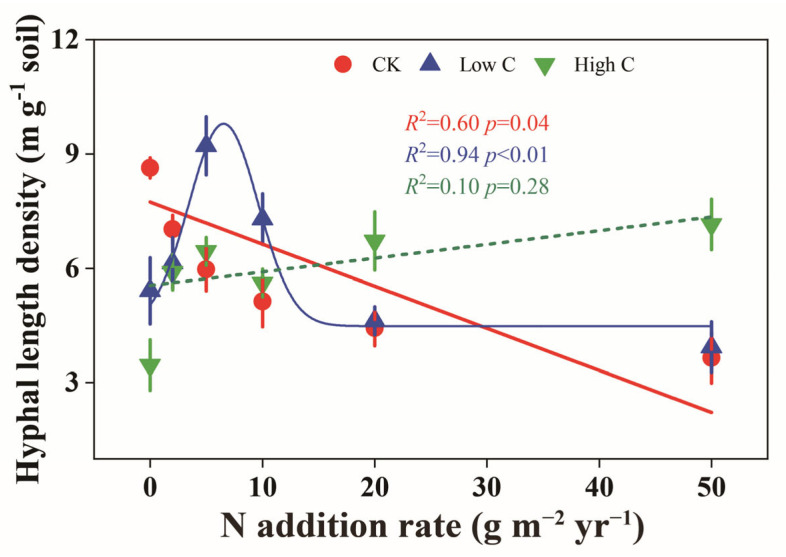
Effects of historical N enrichment and subsequent labile C addition on surface soil (0–10 cm) hyphal length density of arbuscular mycorrhizal fungi. CK represents control treatment with only water added, while Low C and High C correspond to carbon addition levels of 200 and 2000 g C m^−2^ yr^−1^, respectively. Error bars represent ±SE (n = 8). Data were fitted using linear, polynomial or nonlinear curve fitting methods, selecting the most appropriate curve type for each C level.

In control plots, soil HLD exhibited a linear decrease with increasing N addition (R^2^ = 0.60, *p* = 0.04). Under low C addition, soil HLD demonstrated a non-linear response to N levels (R^2^ = 0.94, *p* < 0.01), initially increasing and peaking around 5–10 g N m^−2^ yr^−1^ before sharply declining at higher N levels (N20 and N50). In contrast, high carbon addition led to an increasing trend in soil HLD across the entire N gradient, with a significant increase under high-N conditions (N20 and N50) compared to the control (Figure 3).

### 2.3. Root C, N and P Content and Stoichiometry in Response to Nitrogen and Carbon Treatments

A three-way ANOVA revealed significant main and interactive effects of N enrichment, C addition and plant species on root C, N, P content and stoichiometric ratios (Table S3). Root C content decreased in all species as N levels increased, although this decline was alleviated in *S. baicalensis* and *T. lanceolata* under high C addition (Figure S3b,c). In contrast, *L. chinensis* and *P. bifurca* exhibited more stable root C content under low-N conditions when high C was applied (Figure S3a,d). Across all species, root N content increased significantly with rising N levels, with a linear increase observed in *L. chinensis* and *S. baicalensis* across all C treatments (Figure S3e,f). However, in *T. lanceolata* and *P. bifurca*, root N content increased under control and high-C conditions but remained unchanged under low-C treatment as N addition increased (Figure S3g,h). The response of root P content to N and C treatments varied among species. In *L. chinensis*, root P content decreased under low-N and high-C conditions, while *S. baicalensis* maintained stable P levels across treatments (Figure S3i,j). In *T. lanceolata* and *P. bifurca*, root P content decreased with N addition but stabilized or slightly increased under high-N and high-C or low-C conditions (Figure S3k,l).

**Figure 4:**
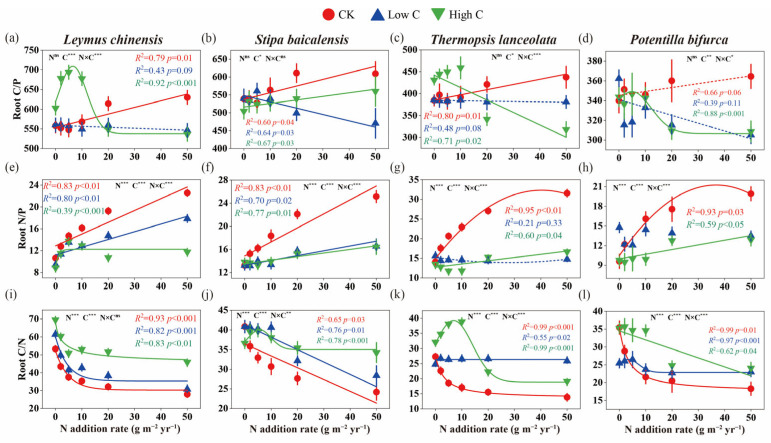
Effects of historical N enrichment and subsequent labile C addition on root C, N and P stoichiometric ratios in different plant species. (**a**–**d**) Root C/P ratio; (**e**–**h**) Root N/P ratio; (**i**–**l**) Root C/N ratio. CK denotes control with only water added; Low C and High C correspond to carbon addition levels of 200 and 2000 g C m^−2^ yr^−1^, respectively. Error bars represent ±SE (n = 8). Data were fitted using linear, polynomial or nonlinear curve fitting methods, selecting the most suitable curve type for each C level.

For *L. chinensis*, high C addition increased root C/P ratio under low-N condition, but decreased root C/P ratio under high-N conditions (Figure 4a). In *S. baicalensis*, the root C/P ratio increased under control and low-C condition with higher N levels, while high C reduced root C/P ratio (Figure 4b). Compared to the control, *T. lanceolata* showed a significant increase in the root C/P ratio under low-N conditions with high C addition, but a significant decrease under high-N conditions (Figure 4c). In *P. bifurca*, root C/P ratio was significantly reduced by C addition (Figure 4d). The root C/N ratio declined sharply across all species as N levels increased, particularly under control and low C treatments. However, under low-C conditions, the root C/N ratio did not change significantly in *T. lanceolata* and *P. bifurca*. High C addition moderated this decline, especially in *L. chinensis* and *S. baicalensis* (Figure 4i–l). The root N/P ratio increased with rising N levels across all species, with the most pronounced effects observed in *L. chinensis* and *S. baicalensis*, where high C mitigated the nitrogen-driven increase in N/P ratio (Figure 4e–h). The N/P ratio of *T. lanceolata* and *P. bifurca* remained stable under low C addition (Figure 4g,h).

### 2.4. Potential Drivers of AMF Infection Metrics in Different Plant Species

**Figure 5:**
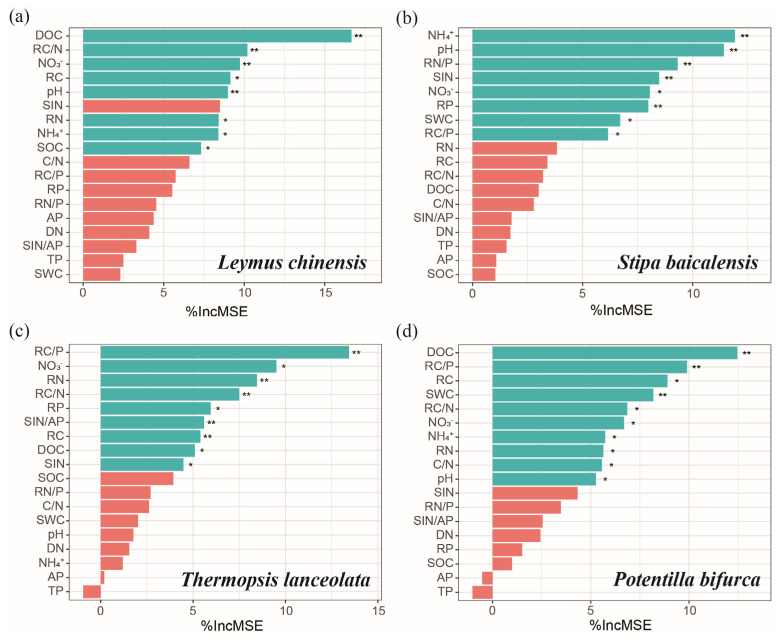
Importance of historical N enrichment and subsequent labile C addition in explaining variance in the first principal component (PC1) of AMF infection metrics across different plant species based on random forest analysis. The plant species represented are (**a**) *L. chinensis* (Trin. ex Bunge) Tzvelev, explaining 66.47% of the variance; (**b**) *S. baicalensis* Roshev., explaining 77.40% of the variance; (**c**) *T. lanceolata* R. Br., explaining 69.51% of the variance; and (**d**) *P. bifurca* Linn., explaining 74.48% of the variance. The importance of each variable is measured as the percentage increase in mean squared error (%IncMSE) when the variable is randomly permuted. NH_4_^+^ indicates soil NH_4_^+^-N; NO_3_^−^ indicates soil NO_3_^−^-N; RC, RN, RP, RC/N, RC/P and RN/P represent root carbon, nitrogen, phosphorus content and their ratios; SIN refers to soil inorganic N; SOC indicates soil organic C; SWC indicates soil water content; DOC indicates soil dissolved organic C; DN indicates soil dissolved N; C/N represents the ratio of DOC to DN; AP indicates soil available P; TP indicates soil total P. Significant variables are marked with asterisks: * *p* < 0.05, ** *p* < 0.01.

Random forest analysis identified key variables contributing to the variance in the first principal component (PC1) of AMF infection metrics for each species (Figure 5). For *L. chinensis*, the most influential variables were dissolved organic carbon (DOC), root C/N ratio, NO_3_^−^-N and pH, all of which had significant effects (*p* < 0.01). Additionally, root C content, root N content, NH_4_^+^-N and soil organic carbon (SOC) also significantly influenced AMF infection metrics (*p* < 0.05) (Figure 5a). In *S. baicalensis*, the most important variables were NH_4_^+^-N, pH, root N/P ratio, soil inorganic nitrogen (SIN) and root P content, each if which contributed significantly to AMF infection metrics (*p* < 0.01). NO_3_^−^-N, soil water content (SWC) and root C/P ratio also had significant effects (*p* < 0.05) (Figure 5b). For *T. lanceolata*, the primary contributors were root C/P ratio, root N content, root C/N ratio, SIN/AP and root C content (*p* < 0.01), with NO_3_^−^-N, root P content, DOC and SIN also showing significant impacts (*p* < 0.05) (Figure 5c). In *P. bifurca*, the most influential variables were DOC, root C/P ratio and SWC (*p* < 0.01). Additionally, root C content, root C/N ratio, NO_3_^−^-N, NH_4_^+^-N, root N content, soil C/N ratio and pH also significantly affected AMF infection metrics (*p* < 0.05) (Figure 5d).

**Figure 6:**
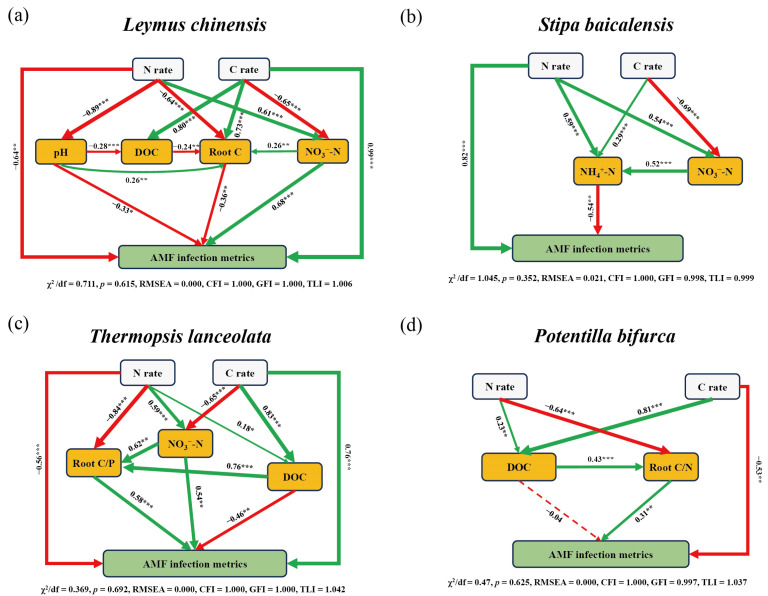
Structural equation models (SEMs) illustrating the relationships between N addition rates, C addition rates, key variables and the first principal component (PC1) of AMF infection metrics across different plant species. The models illustrate the direct and indirect effects of N and C addition on AMF infection metrics through selected variables. (**a**) *L. chinensis* (Trin. ex Bunge) Tzvelev: PC1 explains 66.47% of the variance; (**b**) *S. baicalensis* Roshev.: PC1 explains 77.40% of the variance; (**c**) *T. lanceolata* R. Br.: PC1 explains 69.51% of the variance; (**d**) *P. bifurca* Linn.: PC1 explains 74.48% of the variance. Path coefficients are indicated on each arrow. Green solid lines represent significant positive correlations, while red solid lines represent significant negative correlations (*p* < 0.05). Dotted lines represent non-significant relationships (*p* > 0.05). The thickness of the lines corresponds to the strength of the relationship. Model fit indices at the bottom of each panel indicate a good fit for each SEM (e.g., χ^2^/df, RMSEA, CFI, GFI, TLI). NH_4_^+^-N indicates soil NH_4_^+^-N; NO_3_^−^-N indicates soil NO_3_^−^-N; Root C, Root C/N and Root C/P represent root C, the ratio of root C and N and the ratio of root C and P; DOC indicates soil dissolved organic C. Significance levels are indicated as follows: * *p* < 0.05, ** *p* < 0.01, *** *p* < 0.001.

Structural equation models (SEMs) revealed that AMF infection metrics of each species were influenced directly or indirectly by historical N and C additions (Figure 6). For *L. chinensis*, N addition had a significant direct negative effect on AMF infection metrics (standard estimate = −0.64, *p* < 0.001), whereas C addition significantly increased AMF infection metrics (standard estimate = 0.99, *p* < 0.001). C addition also moderated the positive effect of historical N on soil NO_3_^−^-N (standard estimate = 0.61, *p* < 0.001), thereby indirectly enhancing AMF infection metrics through NO_3_^−^-N (standard estimate = 0.68, *p* > 0.001). Furthermore, C addition mitigated the negative impact of N addition on root C content (standard estimate = −0.64, *p* < 0.001), but the increased root C content subsequently reduced AMF infection metrics (standard estimate = −0.36, *p* < 0.01). Notably, C addition did not alleviate the negative effect of N enrichment on soil pH (standard estimate = −0.89, *p* < 0.001), nor did it modify the negative influence of pH on AMF infection metrics (standard estimate = −0.33, *p* < 0.05) (Figure 6a). In *S. baicalensis*, C addition alleviated the positive impact of N addition on NO_3_^−^-N (standard estimate = 0.54, *p* < 0.001) but did not affect the positive effect of N addition on NH_4_^+^-N (standard estimate = 0.59, *p* < 0.001). The subsequent conversion of NO_3_^−^-N to NH_4_^+^-N ultimately led to a reduction in AMF infection metrics via NH_4_^+^-N (standard estimate = −0.54, *p* < 0.01). In addition, N addition had a direct positive effect on AMF infection metrics (standard estimate = 0.82, *p* < 0.001) (Figure 6b).

In *T. lanceolata*, N addition directly decreased AMF infection metrics (standard estimate = −0.56, *p* < 0.001), while C addition had a direct positive effect on AMF infection metrics (standard estimate = 0.76, *p* < 0.001). C addition alleviated the positive impact of N addition on NO_3_^−^-N (standard estimate = 0.59, *p* < 0.001), thereby indirectly increasing AMF infection metrics (standard estimate = 0.54, *p* < 0.01). Additionally, the mitigation of NO_3_^−^-N by C addition increased the root C/P ratio (standard estimate = 0.62, *p* < 0.01), which in turn promoted AMF infection metrics (standard estimate = 0.58, *p* < 0.001). However, the positive effect of combined C and N addition on DOC indirectly decreased AMF infection metrics (standard estimate = −0.46, *p* < 0.01) (Figure 6c). In *P. bifurca*, C addition directly decreased AMF infection metrics (standard estimate = −0.53, *p* < 0.01). The positive effect of C addition on DOC increased the root C/N ratio (standard estimate = 0.43, *p* < 0.001), whereas N addition decreased the root C/N ratio (standard estimate = −0.64, *p* < 0.001), ultimately leading to an indirect increase in AMF infection metrics (standard estimate = 0.31, *p* < 0.01) through the root C/N ratio (Figure 6d).

## 3. Discussion

**Figure 7:**
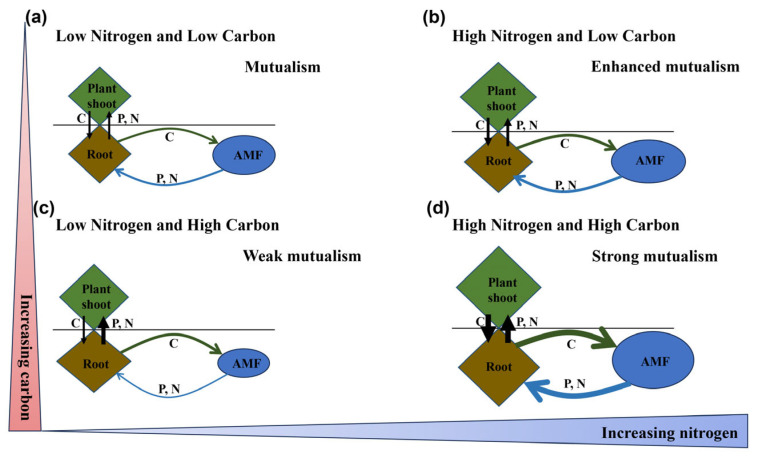
Diagram illustrating the effects of historical N enrichment and C addition on AMF symbiosis. (**a**) Low historical N and low C: A mutualistic symbiosis between host plants and AMF is established through balanced nutrient exchange. Host plants provide C to AMF in exchange for essential minerals. (**b**) High N and low C: Phosphorus becomes limited due to N accumulation. Plants increasingly rely on AMF for P, thereby strengthening the mutualistic relationship. (**c**) The addition of sucrose allowed the plants to increase the amount of carbon transported to the AMF through the mycorrhizal interface without being constrained by the resource of soil nutrients and the relationship became a weak mutualism. (**d**) High N and high C: While nitrogen is abundant, increased C input reduces N availability and exacerbates P limitation. This fosters a strong mutualistic relationship, with AMF playing a crucial role in plant nutrient acquisition. Figure adapted from Ma et al. [13].

Our study provides valuable insights into how C addition mitigates the legacy effects of N addition on AMF symbiosis across four plant species in a temperate grassland. The results reveal complex interactions between historical N and C inputs, indicating C addition can either enhance or diminish AMF–plant relationships, depending on specific N contexts and plant species (Figure 6). Importantly, high C addition under low-N conditions prompted a shift to weak mutualism in *L. chinensis* (Trin. ex Bunge) Tzvelev and *T. lanceolata* R. Br., while high N combined with high-C increased AMF infection in *T. lanceolata* and *P. bifurca* Linn., promoting more robust mutualistic relationships (Figure 7). These findings are crucial for understanding the ecological consequences of N enrichment in grassland ecosystems, as they highlight the intricate interplay between nutrient availability, plant strategy and fungal symbiosis.

### 3.1. Species-Specific Responses to Low N and C Additions

Previous studies have demonstrated that N deposition can significantly alter AMF colonization rates in grassland ecosystems [6]. However, few studies have compared AMF colonization among species from different plant functional groups [43,44], especially under conditions of labile C addition following the cessation of historical N enrichment. A key finding of this study is the pronounced species-specific variation in AMF–plant interactions across different levels of C addition and N legacy. This suggests that plant–AMF interactions are highly context-dependent, shaped by plant nutrient acquisition strategies and specific environmental conditions resulting from N and C inputs, a conclusion consistent with previous research [1,26]. Under low-N and low-C conditions, we observed that *L. chinensis* (perennial rhizomatous grass) and *S. baicalensis* Roshev. (perennial bunchgrass) maintained stable AMF infection density and structural abundance (Figure 1a,b and Figure 2e,f), indicating a stable symbiotic relationship between these grasses and AMF. Previous studies have shown that C addition decreases soil available N [45,46]. Under conditions of low historical N enrichment, low C addition reduced soil N availability, thereby mitigating the negative effects of historical N enrichment on AMF infection metrics (Figure 6a,b).

In contrast, *T. lanceolata* (legume), exhibited a weaker AMF infection density and structural abundance under low-nitrogen and low-carbon conditions (Figure 1c and Figure 2c). Legumes typically form symbiotic relationships with N-fixing bacteria (e.g., rhizobia) to obtain additional N from the soil [47]. Although low C reduced N availability in the soil, N fixation by rhizobia still provided sufficient N for the host plant, reducing the need for AMF to supply N. AMF only provide N to host plants when their own N demands are met [48,49]. Under low-N and low-C conditions, reduced N availability meant that AMF could not supply additional N to *T. lanceolata*. The competition for N between N-fixing bacteria and AMF likely explains the weaker mutualistic relationship observed in *T. lanceolata*. Additionally, AMF are known to supply P, rather than N, as the primary nutrient to host plants [50], suggesting that *T. lanceolata* was not P-limited under these conditions. Since P is relatively immobile in soil [51], it is plausible that *T. lanceolata* was able to acquire sufficient P directly through its root system, without needing AMF assistance.

On the other hand, *P. bifurca* (forb) exhibited higher AMF infection density and structural abundance under low-N and low-C conditions (Figure 1d and Figure 2d,h). As a forb, *P. bifurca* appears to be highly dependent on AMF for nutrient acquisition, showing a stronger mutualistic relationship with AMF under these conditions. Previous studies have shown that N enrichment often enhances the dominance of grasses over forbs in temperate grassland ecosystems [52]. Conversely, AMF can help grasses outcompete forbs under N deposition [53]. In our study, low C addition reduced N availability, which enhanced the competitive advantage of forbs. AMF further facilitated nutrient uptake in *P. bifurca*, reinforcing the competitive position of forbs in these N-limited environments.

### 3.2. Enhanced Mutualism Under High-N and Low-C Conditions

Soil N enrichment is known to hinder grassland restoration, but adding C to the soil can promote recovery by inducing effective N fixation in plants [54]. AMF colonization rates not only differ between grassland plant species [39,43], but the effects of various combinations of historical N and exogenous C additions on AMF infection metrics also vary across species. Unlike the response observed under low-N and low-C conditions, under high-N and low-C conditions, *S. baicalensis* showed a significant increase in AMF infection density and structural abundance (Figure 1b and Figure 2b). During this period, the root C/P ratio of *S. baicalensis* decreased (Figure 4b,f). These findings suggest that AMF play a crucial role in alleviating P limitation in N-rich soils, thereby enhancing the symbiotic relationship between AMF and *S. baicalensis*. Previous studies have confirmed that AMF can alleviate P limitation in plants, enhancing their nutrient absorption, especially P and that C-P exchange rates differ among various symbiotic plants [55].

Contrary to the response observed in *S. baicalensis*, under high-N and low-C conditions, the other three species (*L. chinensis*, *T. lanceolata* and *P. bifurca*) exhibited lower AMF infection density and structural abundance (Figure 1 and Figure 2), resulting in weaker symbiotic relationships with AMF. This may be due to a reduced demand for AMF in these plants in nutrient-rich environments, suggesting that low C addition failed to alleviate the legacy effects of historical N enrichment. Our results further indicate that exogenous C addition directly negatively affected the AMF infection metrics of *P. bifurca* (Figure 6d). Previous research has also shown that AMF colonization rate significantly decrease with C addition, particularly in legumes and forbs, with the effect being more pronounced in N-enriched soils [39]. In AMF–plant symbioses, N transport is only stimulated when C is supplied by the host plant through the mycorrhizal interface and not when C is directly provided to fungal hyphae as an exogenous source [56]. Since the primary C source for AMF comes from the host plant, exogenous C additions do not directly supply C to AMF and thus do not alter AMF growth and reproduction. Overall, exogenous C usually affects AMF infection metrics indirectly, through the stoichiometric relationship between soil and plant root systems.

### 3.3. Weak Mutualism Under Low-N and High-C Conditions

The interactions between plants and AMF are not always mutualistic; they can also become parasitic. Parasitic relationships tend to occur when plant growth is neither N nor P limited [34]. Studies have suggested that nutrient enrichment can drive a shift in mycorrhizal functionality from mutualism to commensalism or even parasitism [13]. In our study, under conditions of low N and high C, *L. chinensis* and *T. lanceolata* exhibited extremely low AMF infection density (Figure 1a,c), as evidenced by the reduced P content in the roots of both species (Figure S3i,k). Under these conditions, there was no significant change in soil HLD compared to the control (Figure 3), which suggests that the relationship between *L. chinensis* and *T. lanceolata* and AMF is weak mutualism. Additionally, the more C that was added, the more NO_3_^−^-N decreased, while light-use efficiency increased proportionally [54]. High C addition enhanced photosynthesis in *L. chinensis* and *T. lanceolata*, allowing them to provide more C to AMF. However, AMF did not reciprocate by providing more nutrients to the host plants, as indicated by the significant increase in the root C/P ratio in both species (Figure 4a,c and Figure 6c). A global meta-analysis has confirmed that soil acidification due to N addition has a stronger influence on AMF dynamics than N availability itself [33]. Our study found that C addition did not alter soil pH (Figure 6a). Although C addition reduced soil NO_3_^−^-N content, AMF infection density in the roots of *L. chinensis* did not increase. Therefore, we hypothesize that soil acidification may have a greater negative impact on AMF infection density in *L. chinensis* roots compared to the effects of increased N availability under low-N and high-C conditions. As a leguminous plant, *T. lanceolata* relies on abundant root nodules to secure its N supply, making it less dependent on AMF.

Interestingly, under low-N and high-C conditions, changes in AMF infection density in the roots of *S. baicalensis* and *P. bifurca* were not significant compared to the control (Figure 1b,d). This suggests that *S. baicalensis* and *P. bifurca* maintained a stable symbiotic relationship with AMF. This stability could be due to the lower competitive ability of *S. baicalensis* and *P. bifurca* for soil nutrients compared to the more dominant species, *L. chinensis* and the N-fixing legume *T. lanceolata*. Thus, *S. baicalensis* and *P. bifurca* may rely more heavily on AMF to obtain nutrients such as N and P. Moreover, AMF diversity has been found to negatively correlate with species-specific responses to N addition and maximum plant height [26]. Since *S. baicalensis* and *P. bifurca* have relatively lower plant heights, they may exhibit more stable AMF diversity, which could explain the consistently stable AMF infection density in their roots.

### 3.4. Strong Mutualism Under High-N and High-C Conditions

High labile C can act as a strong N sink [57]. As hypothesized, under conditions of high N and high C, *T. lanceolata* and *P. bifurca* showed enhanced AMF colonization in their roots, as evidenced by increased vesicle and hyphal abundance (Figure 2c,d,g,h). Our results suggest that C addition alleviated the negative impacts of historical N enrichment on AMF infection metrics in the roots of *T. lanceolata* and *P. bifurca*, leading to a stronger mutualistic relationship between these plants and AMF. Furthermore, both conspecific and heterospecific plants can form common mycorrhizal networks (CMNs), which mediate C and N transfer between plants [58,59]. C addition influences plant composition, with studies showing that sucrose addition leads to increased cover of legumes and forbs [60]. Therefore, high C addition increased the cover of the leguminous plant *T. lanceolata* and the forb *P. bifurca*, thereby enhancing the complexity of the CMNs and the efficiency of nutrient exchange, further strengthening the mutualistic relationships between these plants and AMF. Structural equation modeling showed a significant positive correlation between the root C/P ratio of *T. lanceolata* and the root C/N ratio of *P. bifurca* (Figure 6c,d), indicating that plant stoichiometry plays a key role in promoting AMF symbiosis under high-N and high-C conditions.

However, the effects of C addition on AMF–plant interactions are not uniform across all plant species. C addition has species-specific effects [46]. Sucrose addition has been shown to reduce the cover of grasses compared to legumes and forbs [60]. Under high-N and high-C conditions, *L. chinensis* and *S. baicalensis* exhibited lower AMF infection density and structural abundance in their roots (Figure 1b and Figure 2b,f,j), indicating a weaker mutualistic relationship with AMF. Although AMF colonization was present in the roots of *L. chinensis* and *S. baicalensis*, their symbiotic relationship with AMF was less pronounced compared to that observed in *T. lanceolata* and *P. bifurca*.

These findings emphasize the complexity of AMF–plant interactions under varying nutrient conditions. The ability of C to influence AMF colonization and symbiosis is highly context-dependent, with the effects differing not only by nutrient availability but also by plant species. This highlights the need to consider species-specific traits, plant stoichiometry and the broader ecological context when assessing the role of C in shaping AMF dynamics in ecosystems.

In this study, sucrose is an easily metabolizable carbon source that can stimulate microbial activity in the soil. The addition of sucrose has been shown to enhance nitrogen uptake and transport in AMF–plant symbiosis [56]. However, its effects on microbes and plants can be complex, with both beneficial and potentially adverse consequences. Excessive sucrose input can lead to plant toxicity from manganese and aluminum, which may inhibit plant growth [61]. In addition, sucrose can induce soil microbes to fix nitrogen, reducing the availability of biologically available nitrogen to plants, which may also negatively impact plant growth [62]. Sucrose also affects microbial biomass and community composition. While it does not significantly affect soil pH or exchangeable calcium concentration, it has been shown to increase microbial biomass carbon (MBC) and promote the relative abundance of saprotrophic fungi (SSF) [63]. This shift in microbial community structure can influence soil processes, including organic matter decomposition and soil aggregate formation, both of which play critical roles in soil structure and nutrient cycling [64]. Furthermore, sucrose input can alleviate carbon limitation for saprotrophic fungi in nitrogen-saturated soils, enhancing their activity and promoting soil aggregate stability [63]. Although the addition of sucrose can stimulate microbial activity and support specific microbial communities, its impact on soil microbial dynamics is multifaceted. These changes in microbial biomass and community composition could have cascading effects on nutrient cycling, AMF activity and plant growth. Therefore, future research should carefully consider the potential side effects of sucrose addition, particularly its impact on microbial community structure, when interpreting the results of sucrose-amended soil experiments.

### 3.5. Implications for Grassland Ecosystem Management

The implications of these findings for grassland ecosystem management are significant. The ability of C addition to either enhance or diminish AMF symbiosis, depending on the N context, suggests that managing C inputs could be a viable strategy for modulating plant–AMF interactions in ecosystems experiencing high N deposition. For instance, in regions with high N deposition, targeted C addition could strengthen AMF symbiosis, thereby supporting plant nutrient uptake and enhancing ecosystem resilience [35,46]. Conversely, in low-N environments, careful management of C inputs will be necessary to avoid inadvertently tipping the balance towards weak mutualism, which could undermine the benefits of AMF symbiosis for plant growth and overall ecosystem stability [30,65].

While P availability is a key factor in AMF–plant symbiosis, the potential for P limitation under N and C additions was not directly observed in this study based on the PC1 of AMF infection metrics. But our study revealed that all AMF infection metrics of *L. chinensis* exhibited a significant negative correlation with soil available P (AP), with the exception of the hyphal abundance in roots (Figure S4a). Additionally, the AMF colonization rate and infection intensity in *P. bifurca* also showed a significant negative relationship with AP (Figure S4d). It is crucial to recognize that long-term N enrichment can lead to P limitation in ecosystems, as N inputs can disrupt the natural balance of nutrients [33]. The absence of a direct relationship between P and the PC1 of AMF infection metrics in our findings may be attributed to the short duration of the experiment following the cessation of N addition. Over a longer timescale, P limitation could become more pronounced, potentially altering the dynamics of AMF–plant interactions [30,66,67].

Future studies should explore the potential for long-term P limitation following N enrichment and C addition, particularly as nutrient imbalances can have cascading effects on ecosystem processes. It is also important to consider the indirect effects of N and C on P availability, such as changes in microbial activity and competition. Addressing these interactions will provide a more comprehensive understanding of the long-term impacts of nutrient additions on AMF symbiosis and grassland ecosystem stability.

## 4. Materials and Methods

### 4.1. Study Site

The research was conducted at a temperate meadow steppe (50°10′46.1″N, 119°22′56.4″E) near the Erguna Forest-Steppe Ecotone Research Station, located in Inner Mongolia, China. The region has a cold temperate continental climate, with annual precipitation of 363 mm and a mean temperature of −2.45 °C (1957–2016). The soil is classified as chernozem (FAO), with a pH of 6.8–7.0 in the 0–10 cm topsoil layer. The dominant vegetation includes the C_3_ perennial grasses *Leymus chinensis* (Trin. ex Bunge) Tzvelev and *Stipa baicalensis* Roshev., along with *Artemisia frigida* Willd., *Thermopsis lanceolata* R. Br., *Cymbaria dahurica* L. and *Carex duriuscula* C. A. Mey. which together account for 90% of the total aboveground net primary productivity (ANPP).

### 4.2. Experimental Design

This study relied on the Erguna N compound addition platform, which was established in 2014 using a completely randomized block design and included six N application rates: 0, 2, 5, 10, 20 and 50 g N m^−2^ yr^−1^ (N0, N2, N5, N10, N20 and N50, respectively), equivalent to 0, 20, 50, 100, 200 and 500 kg N ha^−1^ yr^−1^. No fertilizer applications were made before the start of the experiment to ensure that the baseline soil nutrient conditions remained unchanged. The site experiences annual atmospheric N deposition rates of approximately 1–2 g N m^−2^ yr^−1^ [68]. The minimum addition level (2 g N m^−2^ yr^−1^) in this study is close to the ambient N deposition. The higher N addition rates were designed to reflect fertilization or extreme long-term nitrogen deposition [69]. Here, N0 served as the control, with no added nitrogen. Nitrogen treatments were grouped as low-nitrogen (N2–N10) and high nitrogen (N20 and N50). Each treatment was replicated in eight blocks, resulting in 48 plots (10 m × 10 m), spaced 1 m apart with 2 m between blocks [21]. The NH_4_NO_3_ treatment in N fertilizer ceased after 2019, allowing for the assessment of legacy effects.

To explore the influence of labile C addition on AMF colonization after long-term N addition cessation, four 2 m × 2 m subplots were established within each non-mowing NH_4_NO_3_ addition plot, enclosed by iron sheets extending 5 cm above and 20 cm below the ground to minimize lateral nutrient movement. Subplot treatments were: no water or C addition (JCK), water-only control (CK), low C addition (200 g C m^−2^ yr^−1^, Low C) and high C addition (2000 g C m^−2^ yr^−1^, High C). Commercial sucrose (42.1% carbon) was chosen as the labile C source and added evenly five times between June and July in both 2019 and 2020, with each interval lasting two weeks. The high C content and sucrose type consistent with previous research [46]. Each application involved 380.05 g of sucrose for Low C and 3800.5 g for High C, dissolved in 4 L of water and sprayed around plant roots, followed by an additional 4 L rinse to ensure soil infiltration. JCK subplots received no water or sucrose, while CK subplots were treated with 8 L of water. Subsequent data analysis showed no significant differences between JCK and CK treatments, so CK was selected as the water-only control for further analyses.

### 4.3. Field Sampling and Measurement

At the end of August 2020, fine roots (≤2 mm diameter) of four key plant species—*Leymus chinensis*, *Stipa baicalensis*, *Thermopsis lanceolata* and *Potentilla bifurca* Linn.—were carefully excavated for root sampling. These species represent different functional groups: perennial rhizomatous grass, perennial bunchgrass, legume and forb, respectively (detailed species information in Table S2). These species were selected based on their ecological significance in the grassland ecosystem, as they contribute to primary productivity, nitrogen fixation and overall ecosystem stability. Plant samples were collected from randomly selected plants within the study plots. We ensured to avoid the edges of the plots to minimize any edge effects. For each sampled plant, we carefully used a clean small trowel to excavate the entire root system. For plants with fewer fine roots (diameter ≤ 2 mm), we increased the number of plants sampled to ensure an adequate quantity of fine roots for analysis. The collected roots were preserved in 75% ethanol at −20 °C for further analysis. The fine roots were oven-dried at 65 °C for 48 h, pulverized and sieved through a 0.5 mm mesh for chemical analysis. Root C and N contents were determined using an elemental analyzer (Elementar Vario EL III, Elementar Analysen Systeme GmbH, Langenselbold, Germany). Root P content was quantified by Mo-Sb colorimetric spectrophotometry following H_2_SO_4_ + H_2_O_2_ digestion.

Soil samples were collected from each plot using five randomly located cores (3 cm diameter, 0–10 cm depth) and combined into a composite sample. After sieving through a 2 mm mesh, NH_4_^+^-N and NO_3_^−^-N were measured using a continuous-flow ion auto-analyzer (AA3, SEAL Analytical, Norderstedt, Germany). Ten grams of fresh soil was dried for 48 h at 105 °C for determining soil water content (SWC). Dissolved organic C (DOC) and total dissolved N (DN) were measured using a total organic carbon (TOC) analyzer (Dimatec Analysentechnik GmbH, Essen, Germany), with the C/N ratio calculated as DOC/DN. Soil pH was determined in a 1:2.5 soil-water suspension using a pH meter (Thermo Fisher Scientific, Waltham, MA, USA). Total P (TP) content in soil is determined using the H_2_SO_4_-HClO_4_ digestion method. Available P (AP) was determined by molybdenum-antimony resistance colorimetry. The color rendering was determined with an ultraviolet Visible Spectrophotometer (UV-2550, UV-Visible spectrophotometer, Shimadzu, Japan). Soil organic C (SOC) was quantified using an external heating method with potassium dichromate [70].

### 4.4. AM Fungal Infection Metrics and Soil Hyphal Length Density

Root samples preserved in 75% ethanol were rinsed with distilled water to remove ethanol and soil particles. The roots were cut into approximately 1 cm segments to facilitate uniform clearing and staining. These segments were immersed in 10% (*w*/*v*) potassium hydroxide (KOH) solution and heated at 60 °C for 60 min to break down cell walls and cellular contents, making fungal structures more visible. After clearing, the root segments were acidified in 5% (*v*/*v*) acetic acid for 30 min to neutralize any remaining KOH and prepare the roots for staining. The acidified root segments were immersed in a solution of 5% acetic acid ink (Parker pure black writing ink) for staining and heated at 60 °C for 30 min [71]. Following staining, the root segments were decolorized in water for 12 h to remove excess stain and improve visibility of internal fungal structures, ensuring that AMF colonization could be clearly observed. 30 root segments per sample in each replicate were chosen for the assessment of mycorrhizal colonization. The AMF infection metrics quantified include: AMF colonization rate, infection density, vesicular abundance (V%), hyphal abundance (H%) and arbuscular abundance (A%). These AMF infection metrics were calculated using the MYCOCALC software (https://www2.dijon.inrae.fr/mychintec/Mycocalc-prg/download.html (accessed on 29 July 2023)) [72]. The calculation of V% and H% is the same as A%.

Soil hyphal length density was assessed by extracting AMF hyphae from soil samples. Approximately 5 g of fresh soil per sample was processed using the method described by Brundrett et al. [73]. The soil was first suspended in a water solution, stirred thoroughly and then poured through a 45 µm mesh to separate hyphal fragments from larger soil particles. The retained material was then stained with trypan blue, which selectively binds to fungal hyphae, providing a clear contrast against non-fungal structures. The AMF hyphae were differentiated from other types of fungal hyphae based on morphological characteristics and their specific reaction to trypan blue staining [74]. The hyphal length density was measured using the grid-line intersection method [73]. The total length of AMF hyphae per gram of soil is then calculated from the intersection counts, providing an estimate of the density of AMF hyphal networks in the soil.

### 4.5. Statistical Analysis

The effects of historical N enrichment, subsequent labile C addition and plant species on AMF infection metrics—including AMF colonization rate, infection density, vesicular abundance, hyphal abundance and arbuscular abundance—were assessed using three-way analysis of variance (ANOVA). The ANOVA factors included N addition, C addition, plant species and their interactions. Significant differences between treatments were examined using Tukey’s Honest Significant Difference (HSD) test. Linear and nonlinear regression analyses were employed to explore relationships between N addition and AMF metrics in different C addition levels. Principal component analysis (PCA) summarized multivariate data on AMF infection metrics, with the first principal component (PC1) accounting for a substantial proportion of variance (over 65% for each species; Table S1). Scree plots confirmed the variance captured by PC1 (Figure S1). Random forest analysis identified key factors influencing AMF infection metrics’ PC1 using the *randomForest* package in R [75]. The model’s predictive accuracy was evaluated based on the percentage increase in mean squared error (%IncMSE). A structural equation model (SEM) was constructed to quantify direct and indirect effects of N and C additions on AMF infection metrics, incorporating soil properties and plant root nutrient parameters as mediators [76]. Model fit was evaluated using chi-square tests (*p* > 0.05), CFI (>0.95) and RMSEA (<0.05) [77,78]. Data visualization was primarily conducted using the *ggplot2* package in R, with regression analyses visualized using Origin 2018. All statistical analyses were performed using R software (version 4.3.0) and SPSS 26.

## 5. Conclusions

Our study reveals species-specific responses to long-term N enrichment and C addition in the AMF symbiosis within a temperate grassland ecosystem. *L. chinensis* (Trin. ex Bunge) Tzvelev and *T. lanceolata* R. Br. exhibited decreased AMF infection metrics under low-N and high-C conditions, AMF and plants became weak mutualism. In contrast, *S. baicalensis* Roshev. and *P. bifurca* Linn. maintained stable AMF infection metrics, beneficial associations with AMF under similar nutrient conditions. Under high N and C inputs, *T. lanceolata* and *P. bifurca* experienced increased AMF infection, with plant root stoichiometry playing a key role in supporting these mutualistic interactions. These results underscore the complex interactions between nutrient availability and AMF symbiosis, highlighting the necessity of refined management strategies for grassland ecosystems facing global environmental changes. Future research should explore the long-term effects of nutrient dynamics on ecosystem stability, plant community structure and the broader implications for resilience in grassland ecosystems under changing environmental conditions.

## Figures and Tables

**Table 1:** Three-way ANOVA results for arbuscular mycorrhizal fungi (AMF) infection metrics across four plant species under varying N and C addition rates.

Factors	*df*	AMF Colonization Rate		AMF Infection Density		Vesicular Abundance		Hyphal Abundance		Arbuscular Abundance	
		*F*	*p*	*F*	*p*	*F*	*p*	*F*	*p*	*F*	*p*
Plant	3	136.39	**<0.001**	64.23	**<0.001**	24.90	**<0.001**	7.87	**<0.001**	23.45	**<0.001**
N	5	14.76	**<0.001**	30.23	**<0.001**	22.15	**<0.001**	14.52	**<0.001**	23.97	**<0.001**
C	2	37.63	**<0.001**	1.20	0.302	14.53	**<0.001**	8.31	**<0.001**	8.05	**<0.001**
Plant × N	15	11.92	**<0.001**	16.68	**<0.001**	11.50	**<0.001**	6.13	**<0.001**	18.54	**<0.001**
Plant × C	6	54.00	**<0.001**	49.80	**<0.001**	26.27	**<0.001**	18.60	**<0.001**	17.30	**<0.001**
N × C	10	77.95	**<0.001**	77.02	**<0.001**	33.87	**<0.001**	23.78	**<0.001**	33.09	**<0.001**
Plant × N × C	29	15.52	**<0.001**	19.54	**<0.001**	15.77	**<0.001**	5.60	**<0.001**	13.71	**<0.001**

Significant results are indicated in bold (*p* < 0.01). Plant refers to the four species studied (*L. chinensis* (Trin. ex Bunge) Tzvelev, *S. baicalensis* Roshev., *T. lanceolata* R. Br. and *P. bifurca* Linn.); N denotes different nitrogen addition rates (0, 2, 5, 10, 20 and 50 g N m^−2^ yr^−1^); C indicates carbon addition rates (control (water only), 200 g C m^−2^ yr^−1^ and 2000 g C m^−2^ yr^−1^).

**Table 2:** Two-way ANOVA results for surface soil hyphal length density of arbuscular mycorrhizal fungi (AMF) under different N and C addition rates.

Factors	*df*	Hyphal Length Density	
		*F*	*p*
N	5	47.92	**<0.001**
C	2	3.49	**0.03**
N × C	10	81.29	**<0.001**

Significant results are indicated in bold (*p* < 0.05). N represents various nitrogen addition rates (0, 2, 5, 10, 20 and 50 g N m^−2^ yr^−1^); C denotes carbon addition rates (control (water only), 200 g C m^−2^ yr^−1^ and 2000 g C m^−2^ yr^−1^).

## Data Availability

Data is contained within the article.

## Supplementary Materials

The following supporting information can be downloaded at: https://www.mdpi.com/article/10.3390/plants14030456/s1, Figure S1: Scree plots from the PCA of AMF infection metrics of different plant species; Figure S2: Effects of historical N enrichment and subsequent labile C addition on AMF colonization rate of plant roots across different plant species; Figure S3: Effects of historical N enrichment and subsequent labile C addition on root C, N, and P content in different plants; Figure S4: Correlations between five AMF infection metrics of the roots of four key plant species and related soil properties, as well as the C, N, and P content in plant roots; Table S1: The variance contribution rates of PC1–PC5 derived from the PCA analysis of four different plant species; Table S2: Under conditions of historical N enrichment and labile C addition, root samples from four plant species were collected across different plots to assess AMF infection metrics; Table S3: Three-way ANOVA for root C, N, P content and stoichiometric ratios of four plants in different N and C addition rates.
