# Supplementary material for: Labile Carbon Input Mitigates the Negative Legacy Effects of Nitrogen Addition on Arbuscular Mycorrhizal Symbiosis in a Temperate Grassland
Source: Plants (Basel). 2025 Feb 4;14(3):456. doi: 10.3390/plants14030456 (PMC11820778; doi:10.3390/plants14030456)
Supplement: Supplementary file 1 [file plants-14-00456-s001.zip › plants-3395983-supplementary.pdf]

Supplementary Material

Fig. S1 Scree plots from the principal component analysis (PCA) of arbuscular mycorrhizal fungi (AMF) infection metrics of plant roots across different plant species, showing the variance explained by the first principal component (PC1). The proportion of variance captured by PC1 is as follows: (a) *Leymus chinensis* – 66.47%; (b) *Stipa baicalensis* – 77.40%; (c) *Thermopsis lanceolata* – 69.51%; and (d) *Potentilla bifurca* – 74.48%.

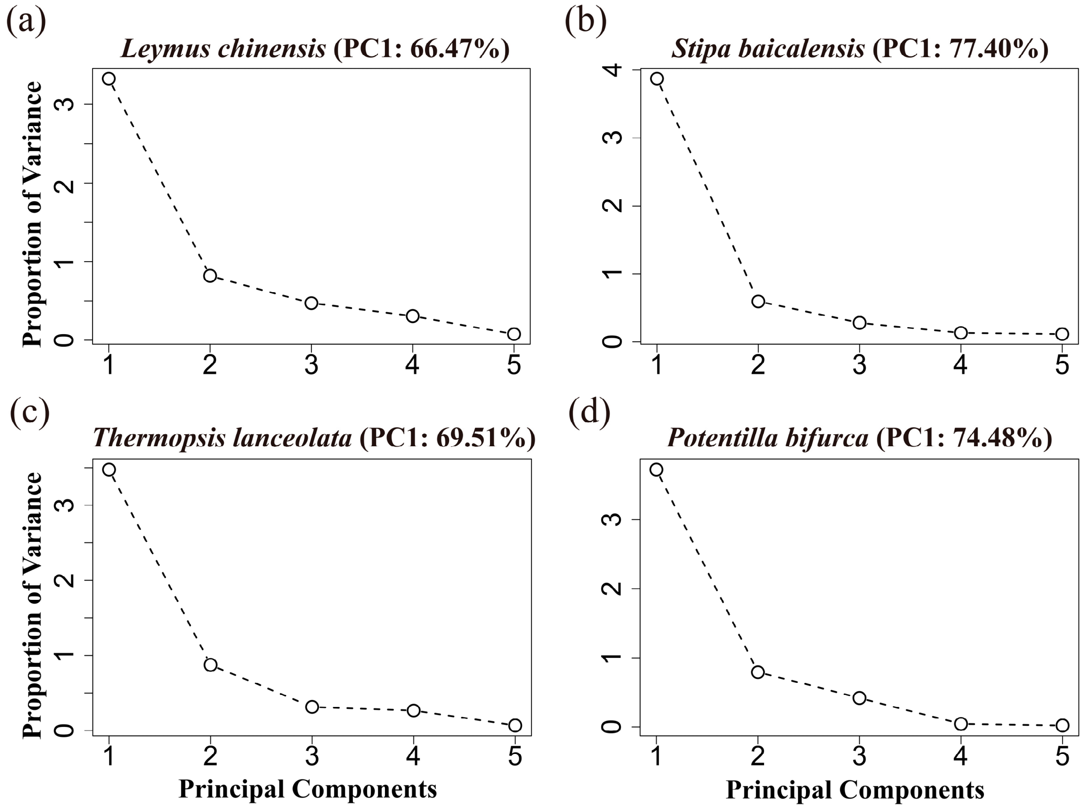

Fig. S2 Effects of historical N enrichment and subsequent labile C addition on arbuscular mycorrhizal fungi (AMF) colonization rate of plant roots across different plant species. (a) *Leymus chinensis*; (b) *Stipa baicalensis*; (c) *Thermopsis lanceolata*. (d) *Potentilla bifurca*. CK represents control treatment with only water added, while Low C and High C represent carbon addition levels of 200 and 2000 g C m^{-2} yr^{-1}, respectively. Error bars represent ±SE (n = 8). Data were fitted using linear, polynomial, or nonlinear curve fitting methods, with the most appropriate curve type selected for each C level.

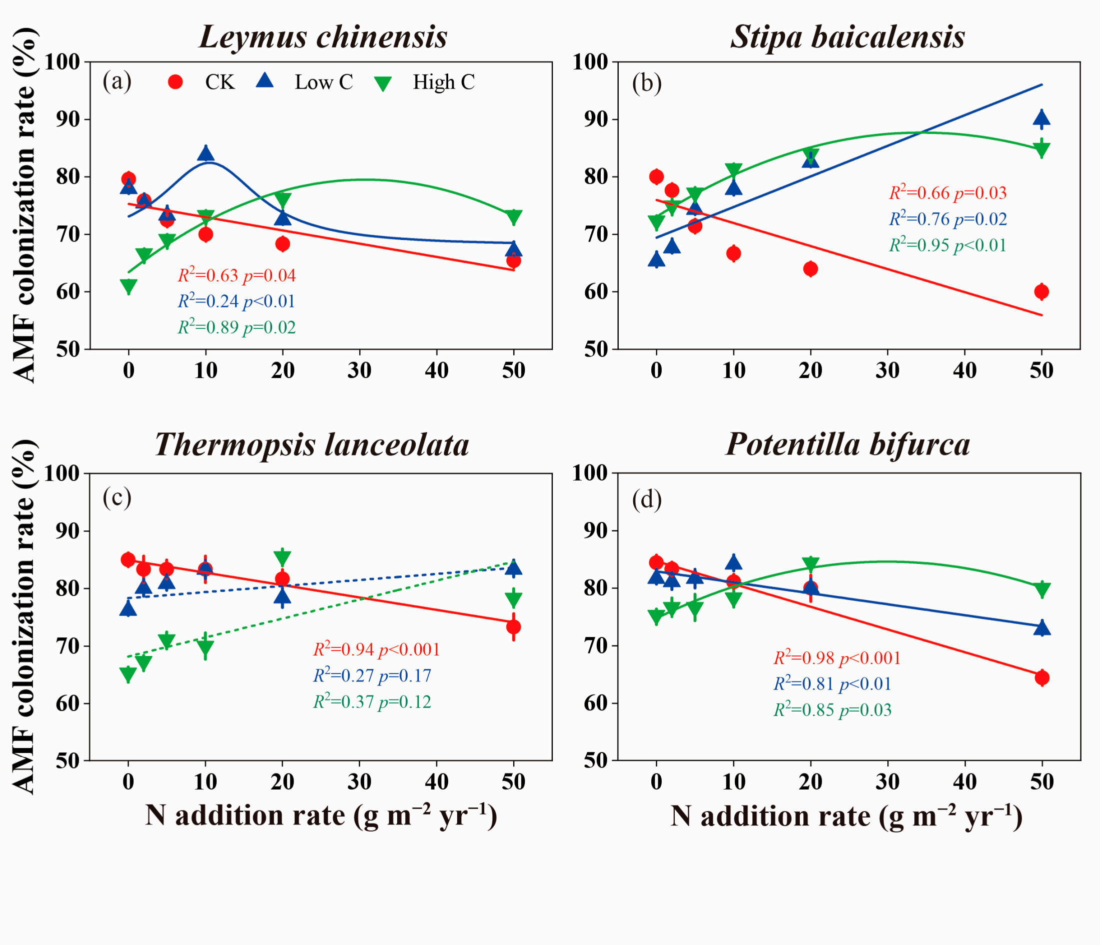

Fig. S3 Effects of historical N enrichment and subsequent labile C addition on root C, N, and P content in different plants. (a-d) Root C content; (e-h) Root N content; (i-l) Root P content. CK represents control treatment with only water added, while Low C and High C represent C addition levels of 200 and 2000 g C m^{-2} yr^{-1}, respectively. Error bars represent ±SE (n = 8). Data were fitted using linear, polynomial, or nonlinear curve fitting methods, with the most appropriate curve type selected for each C level.

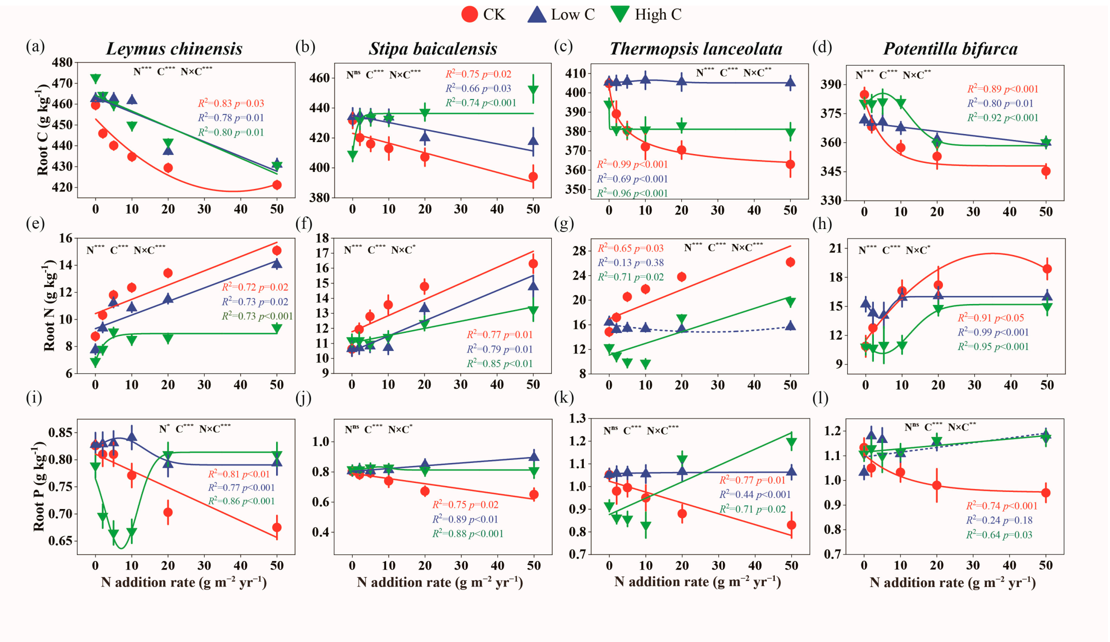

Fig. S4 Correlations between five arbuscular mycorrhizal fungal (AMF) infection metrics of the roots of four key plant species and related soil properties, as well as the C, N, and P content in plant roots. (a) *Leymus chinensis*; (b) *Stipa baicalensis*; (c) *Thermopsis lanceolata*. (d) *Potentilla bifurca*. RC, RN, RP, RC/N, RC/P, and RN/P represent root carbon, nitrogen, phosphorus content and the ratio of them; AP, soil available P; TP, soil total P; DOC, soil dissolved organic C; DN, soil dissolved N; C/N, the ratio of DOC to DN; NH_4^+, soil NH_4^+-N; NO_3^-, soil NO_3^--N; SIN, soil inorganic N; SOC indicates soil organic C; SWC indicates soil water content; F, AMF colonization rate; M, AMF infection density; V, vesicular abundance; H, hyphal abundance; A, arbuscular abundance. The color blue designates a positive correlation, the red a negative correlation. The level of correlations was illustrated by color intensity and relative size of the square marks. The levels of statistical significance were labeled using asterisk (*, **, *** represent $P < 0.05$, 0.01, and 0.001, respectively).

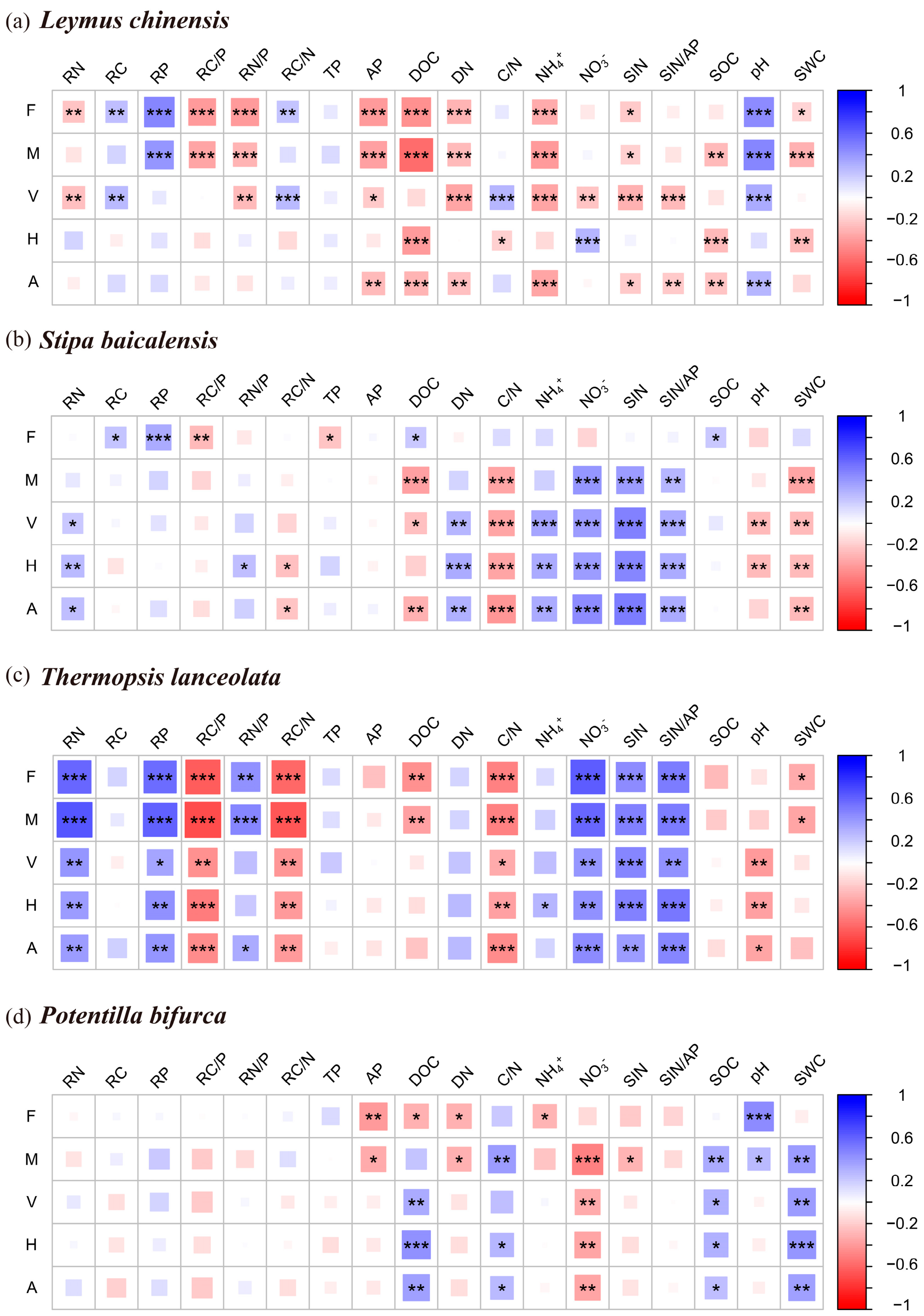
(a) Leymus chinensis
(b) Stipa baicalensis
(c) Thermopsis lanceolata
(d) Potentilla bifurca
RN
RC
RP
RC/P
RN/P
RC/N
TP
AP
DOC
DN
C/N
NH_4^+
NO_3^-
SIN
SIN/AP
SOC
pH
SWC
F
M
V
H
A
1
0.6
0.2
−0.2
−0.6
−1

Table S1 The variance contribution rates of the five principal components (PC1–PC5) derived from the PCA analysis of four different plant species, summarizing the percentage of total variance explained by each component.

**Plant species**	**PC1**	**PC2**	**PC3**	**PC4**	**PC5**
Leymus chinensis	66.47%	16.38%	9.43%	6.16%	1.57%
Stipa baicalensis	77.40%	11.91%	5.66%	2.69%	2.33%
Thermopsis lanceolata	69.51%	17.51%	6.31%	5.34%	1.32%
Potentilla bifurca	74.48%	15.88%	8.39%	0.85%	0.40%

Table S2 Under conditions of historical N enrichment and labile C addition, root samples from four plant species were collected across different plots to assess AMF infection metrics.

N	C	*Leymus chinensis*	*Stipa baicalensis*	*Thermopsis lanceolata*	*Potentilla bifurca*
**0**	**High C**	1	0	1	1
**0**	**High C**	1	1	1	0
**0**	**High C**	1	1	0	1
**0**	**High C**	1	1	0	0
**0**	**High C**	1	1	0	0
**0**	**High C**	1	1	1	1
**0**	**High C**	1	1	1	1
**0**	**High C**	1	1	1	1
**0**	**Low C**	1	0	1	1
**0**	**Low C**	1	0	1	0
**0**	**Low C**	1	0	1	1
**0**	**Low C**	1	1	1	0
**0**	**Low C**	1	1	1	1
**0**	**Low C**	1	1	1	1
**0**	**Low C**	1	1	0	1
**0**	**Low C**	1	1	1	1
**0**	**CK**	1	1	1	0
**0**	**CK**	1	0	0	0
**0**	**CK**	1	0	1	0
**0**	**CK**	1	1	0	0
**0**	**CK**	1	1	1	0
**0**	**CK**	1	1	0	1
**0**	**CK**	1	1	0	1
**0**	**CK**	1	1	1	1
**2**	**High C**	1	0	0	0
**2**	**High C**	1	0	1	0
**2**	**High C**	1	0	1	1
**2**	**High C**	1	0	1	0
**2**	**High C**	1	1	0	0
**2**	**High C**	1	1	1	0
**2**	**High C**	1	1	1	0
**2**	**High C**	1	1	0	1
**2**	**Low C**	1	1	1	0
**2**	**Low C**	1	1	1	0
**2**	**Low C**	1	0	0	1

**2**	**Low C**	1	1	0	0
**2**	**Low C**	1	1	0	0
**2**	**Low C**	1	1	0	0
**2**	**Low C**	1	1	1	1
**2**	**Low C**	1	1	0	1
**2**	**CK**	1	1	1	0
**2**	**CK**	1	1	0	1
**2**	**CK**	1	0	0	1
**2**	**CK**	1	1	0	0
**2**	**CK**	1	1	0	0
**2**	**CK**	1	1	0	1
**2**	**CK**	1	1	0	0
**2**	**CK**	1	1	0	1
**5**	**High C**	1	1	1	0
**5**	**High C**	1	1	0	0
**5**	**High C**	1	1	0	0
**5**	**High C**	1	1	1	0
**5**	**High C**	1	0	1	0
**5**	**High C**	1	0	0	0
**5**	**High C**	1	1	0	0
**5**	**High C**	1	1	0	1
**5**	**Low C**	1	0	1	0
**5**	**Low C**	1	1	0	0
**5**	**Low C**	1	1	1	0
**5**	**Low C**	1	1	1	0
**5**	**Low C**	1	1	0	0
**5**	**Low C**	1	1	0	1
**5**	**Low C**	1	1	0	0
**5**	**Low C**	1	1	1	1
**5**	**CK**	1	1	0	0
**5**	**CK**	1	1	0	0
**5**	**CK**	1	1	0	0
**5**	**CK**	1	1	1	0
**5**	**CK**	1	1	0	0
**5**	**CK**	1	0	0	0
**5**	**CK**	1	1	0	0
**5**	**CK**	1	1	1	0
**10**	**High C**	1	1	0	0
**10**	**High C**	1	1	0	0
**10**	**High C**	1	1	0	1
**10**	**High C**	1	1	1	1
**10**	**High C**	1	0	0	1
**10**	**High C**	1	1	0	1
**10**	**High C**	1	1	0	0

**10**	**High C**	1	1	0	0
**10**	**Low C**	1	1	0	0
**10**	**Low C**	1	1	1	0
**10**	**Low C**	1	1	0	1
**10**	**Low C**	1	1	0	1
**10**	**Low C**	1	0	0	0
**10**	**Low C**	1	1	0	1
**10**	**Low C**	1	1	1	1
**10**	**Low C**	1	0	0	0
**10**	**CK**	1	0	0	0
**10**	**CK**	1	1	0	0
**10**	**CK**	1	0	0	1
**10**	**CK**	1	0	1	1
**10**	**CK**	1	0	0	0
**10**	**CK**	1	0	0	1
**10**	**CK**	1	1	0	0
**10**	**CK**	1	1	0	0
**20**	**High C**	1	1	0	0
**20**	**High C**	1	0	0	1
**20**	**High C**	1	0	0	0
**20**	**High C**	1	1	1	1
**20**	**High C**	1	0	1	1
**20**	**High C**	1	1	0	1
**20**	**High C**	1	1	1	1
**20**	**High C**	1	1	0	1
**20**	**Low C**	1	1	0	0
**20**	**Low C**	1	1	0	0
**20**	**Low C**	1	1	0	0
**20**	**Low C**	1	1	0	1
**20**	**Low C**	1	1	0	0
**20**	**Low C**	1	1	0	1
**20**	**Low C**	1	1	1	1
**20**	**Low C**	1	1	1	1
**20**	**CK**	1	1	0	0
**20**	**CK**	1	1	0	0
**20**	**CK**	1	1	0	0
**20**	**CK**	1	1	0	1
**20**	**CK**	1	0	0	0
**20**	**CK**	1	1	1	0
**20**	**CK**	1	0	1	0
**20**	**CK**	1	0	0	0
**50**	**High C**	1	0	1	0
**50**	**High C**	1	0	1	0
**50**	**High C**	1	0	0	1

**50**	**High C**	1	1	0	0
**50**	**High C**	1	0	0	0
**50**	**High C**	1	0	0	1
**50**	**High C**	1	1	0	1
**50**	**High C**	1	0	0	1
**50**	**Low C**	1	1	0	0
**50**	**Low C**	1	0	0	1
**50**	**Low C**	1	0	0	1
**50**	**Low C**	1	1	1	1
**50**	**Low C**	1	0	1	0
**50**	**Low C**	1	0	1	1
**50**	**Low C**	1	0	0	1
**50**	**Low C**	1	0	0	1
**50**	**CK**	1	0	1	0
**50**	**CK**	1	1	0	0
**50**	**CK**	1	1	0	0
**50**	**CK**	1	0	0	1
**50**	**CK**	1	0	0	0
**50**	**CK**	1	0	0	1
**50**	**CK**	1	0	0	1
**50**	**CK**	1	1	0	0

Value of 0 indicates no root samples were collected, while 1 indicates successful sample collection. N represents different nitrogen addition rates (0, 2, 5, 10, 20 and 50 g N m^{-2} yr^{-1}); C represents different carbon addition rates (CK (only water addition without C), 200 g C m^{-2} yr^{-1} and 2000 g C m^{-2} yr^{-1}).

Table S3 Three-way ANOVA for root C, N, P content and stoichiometric ratios of four plants in different N and C addition rates. Significant results are in bold ($p < 0.01$). Plant represents the four plants (*L. chinensis*, *S. baicalensis*, *T. lanceolata* and *P. bifurca*); N represents different nitrogen addition rates (0, 2, 5, 10, 20 and 50 g N m^{-2} yr^{-1}); C represents different carbon addition rates (control (only water addition without C), 200 g C m^{-2} yr^{-1} and 2000 g C m^{-2} yr^{-1}).

Factors	*df*	Root N content		Root C content		Root P content		Root C/P		Root N/P		Root C/N	
		F	*P*	*F*	*P*	*F*	*P*	*F*	*P*	*F*	*P*	*F*	*P*
Plant	3	340.48	**<0.001**	1251.57	**<0.001**	332.56	**<0.001**	392.60	**<0.001**	57.97	**<0.001**	392.99	**<0.001**
N	5	109.16	**<0.001**	40.19	**<0.001**	1.20	0.31	0.73	0.60	72.83	**<0.001**	68.56	**<0.001**
C	2	205.86	**<0.001**	66.49	**<0.001**	34.32	**<0.001**	8.93	**<0.001**	169.95	**<0.001**	110.23	**<0.001**
Plant × N	15	3.66	**<0.001**	6.42	**<0.001**	1.07	0.39	0.72	0.77	2.78	**<0.001**	11.77	**<0.001**
Plant × C	6	23.17	**<0.001**	12.21	**<0.001**	5.72	**<0.001**	5.38	**<0.001**	10.36	**<0.001**	17.87	**<0.001**
N × C	10	14.52	**<0.001**	7.73	**<0.001**	11.96	**<0.001**	5.20	**<0.001**	20.47	**<0.001**	3.67	**<0.001**
Plant × N × C	29	6.44	**<0.001**	4.27	**<0.001**	2.18	**0.001**	1.77	**0.01**	3.40	**<0.001**	2.57	**<0.001**
